# Treating acute fatty liver of pregnancy with artificial liver support therapy: Systematic review

**DOI:** 10.1097/MD.0000000000012473

**Published:** 2018-09-21

**Authors:** Zhenping Wu, Peng Huang, Yi Gong, Junhui Wan, Wei Zou

**Affiliations:** a Department of Infectious Diseases, The First Affiliated Hospital of Nanchang University; b Department of Epidemiology, School of Public Health, Nanchang University; c Department of Gynecology and Obstetrics, The First Affiliated Hospital of Nanchang University, Jiangxi, China.

**Keywords:** acute fatty liver of pregnancy, artificial liver support therapy, disease outcome, multiple organ dysfunctions

## Abstract

**Background:**

Acute fatty liver of pregnancy (AFLP) is a serious disease with high mortality. Recent data suggest that postpartum application of artificial liver support therapy (ALST) in AFLP management improves disease outcome. In current study, we tend to further evaluate whether the type, the timing, and the number of postpartum ALST sessions and other clinical factors including complications and liver and renal function will influence disease outcome in ALST era.

**Methods:**

Medical records of 15 AFLP patients having postpartum ALST in our hospital from 2010 to 2016 were first retrospectively reviewed. A systematic review and statistical analysis was then conducted with our cases and those having postpartum ALST reported in PubMed, Embase, Cochrane, and ClinicalTrials.gov till the end of June 2016.

**Results:**

Nine relevant studies were identified in the public databases. A total of 104 cases were enrolled for analysis including 15 cases from our hospital and 89 cases from the identified 9 studies. Univariate analysis revealed that 2 complications, postpartum hemorrhage and multiple organ dysfunctions (MODS), were associated with the outcome of AFLP patients undergoing postpartum ALST. Binary logistic regression analysis further indicated that MODS was the only independent factor affecting disease outcome. Surprisingly, factors including pre-ALST serum levels of total bilirubin and creatinine, the type and number of postpartum ALST sessions, time interval between delivery and ALST were unrelated to disease outcome.

**Conclusion:**

Current data suggest that postpartum ALST improves the outcome of AFLP patients but more properly designed experiments are needed to confirm this point. Our study further indicates that occurrence of MODS carries a bad prognosis even in ALST era. Our study provides valuable information on guiding the clinical application of postpartum ALST in AFLP management.

## Abstract 2

Supplemental Digital Content is available in the text

## 1 Introduction

Acute fatty liver of pregnancy (AFLP), first described by Sheehan, is a rare but serious disease with an incidence of 1 per 7000 to 16,000 pregnancies. It mostly occurs in the third trimester of pregnancy or during early postpartum period.^[1]^ Prompt recognition of the disease and early termination of pregnancy is essential to improve the overall outcome of both mothers and infants.

So far the pathogenesis of AFLP is not yet completely elucidated, but it is suggested that abnormal β-oxidation of fatty acids in fetal mitochondria that probably is caused by a genetic mutation in long-chain 3-hydroxyl coenzyme A dehydrogenase contributes to microvesicular fatty infiltration of the liver of mothers.^[2]^ Besides, other potential risk factors are also suggested by some studies including primipara, male fetus, and multiparous women.^[3]^

In recent years, mortality of AFLP for both suffering mothers and infants has dramatically decreased due to better understanding of the disease and the implementation of new treatment modality, the artificial liver support therapy (ALST),^[1,4–13]^ in clinic that includes plasma exchange (PE) and molecular adsorbent recycling system (MARS), etc. Since the first reported application of postpartum ALST in 1989 to treat a patient with advanced AFLP, ALST has been used as a postpartum therapy together with other liver-protective treatment methods for AFLP management.^[11]^ The effect of ALST is thought to occur by removal of circulating endotoxins, replacement of normal coagulation factors and proteins, interruption of coagulopathy, and finally improvement of liver function. Importantly, ALST as a form of short-term bridging treatment for nonpregnant patients with acute hepatic failure has been used for some years to allow liver regeneration or to transition between hepatic failure and orthotopic liver transplantation.^[14,15]^ However, in clinical practice certain key questions regarding postpartum ALST in AFLP management still remain to be addressed, which include: When should ALST start after delivery? Is the type of ALST important? How many sessions of ALST should be carried out? Are there any other factors that may potentially influence disease outcome (recovered or deceased)? To study these questions, in this review we will first discuss 15 AFLP cases admitted into our hospital between the year of 2010 and 2016, and then performed a systematic review on the AFLP cases of ours and those reported in the public databases.

## 2 Patients and methods

### 2.1 Description of the 15 cases from our hospital

After approval by the review board of medical ethics of the First Affiliated Hospital of Nanchang University, medical records of 15 patients with the diagnosis of AFLP from 2010 to 2016 were reviewed. Diagnosis of AFLP for each patient was made through the combination of clinical, lab, and imaging findings, which were in accordant to the Swansea criteria.^[16]^ As AFLP is a potentially fatal disease, all the patients diagnosed with AFLP had postpartum ALST. Depending on the disease status when the decision of starting ALST was made, all the patients received PE or MARS, or the combination of PE + continuous venovenous hemofiltration (CVVH) or MARS + hemodialysis.

### 2.2 Strategy of searching and selecting relevant studies from public databases

The systematic review was conducted according to the PRISMA guidelines. A search was conducted in PubMed, Embase, Cochrane, and ClinicalTrials.gov in June 2016 using “acute fatty liver of pregnancy,” “pregnancy-related liver diseases,” or “fatty liver and pregnancy” as the searching keywords. Two investigators independently screened the titles, abstracts, and full texts for study selection and data collection. A third investigator was consulted whenever needed. Inclusion criteria were studies involving patients diagnosed with AFLP and treated with conventional methods plus ALST, and being published after 2006. Non-English publications were excluded. Year of 2006 was selected because this was the time when ALST, after several years’ development, became a more mature and routine method in clinic for the management of liver failure caused by various etiologies. Besides above public databases, reference lists and citations of the included studies were also manually searched to identify potentially eligible studies. Case–control studies, case reports, and retrospective cohort studies were included in the review. No randomized controlled trials or prospective cohort studies were found.

To avoid duplication of included studies and collected data, names of authors and institutions and publishing dates of selected studies were compared. If relevant information we needed could not be obtained or derived from published data, attempt would be made to contact corresponding authors, but unfortunately no corresponding authors of those studies responded to our requests. These studies include those by Yu et al, Chu et al, Martin et al, Tang et al, and Naeyer et al.^[4,5,11,13,17]^ Data without complete information that could not be acquired from corresponding authors were excluded. Following data were extracted from selected studies that met the inclusion criteria: year of publication, number of patients in a single study, patient ages, gestational weeks, delivery method, maternal complications, time interval between delivery and ALST, type of and number of ALST sessions implemented, and fetal and maternal outcomes.

### 2.3 Statistical analysis

Statistical review of the study was performed by a biomedical statistician who is also one of the first authors of this manuscript. We first described the characteristics of all the cases both from our hospital and those retrieved from the databases. Quantitative variables that were normally distributed are expressed as mean ± standard deviation, and those that were abnormally distributed are expressed as median (interquartile range). We then examined the factors that may potentially influence disease outcome (recovered or deceased) with single variable or multivariate analysis. Quantitative variables were compared using either the Student *t* test or the rank-sum test. Qualitative variables were expressed as percentages and compared using either the chi-squared test or Fisher exact test. Binary logistic regression was used for the multivariate analysis.

## 3 Results

### 3.1 Characteristics of the cases from our hospital

The mean age of these 15 patients from our hospital was 25.6 years old (range 19–39), and their mean gestational weeks were 35.7 weeks (range 28–40.4). Most of the patients were primigravidas. Three patients had vaginal deliveries and the rest had cesarean sections. There were 16 fetuses born in total with 2 dead and another 3 having unknown fates (Table 1).

**Table 1:**
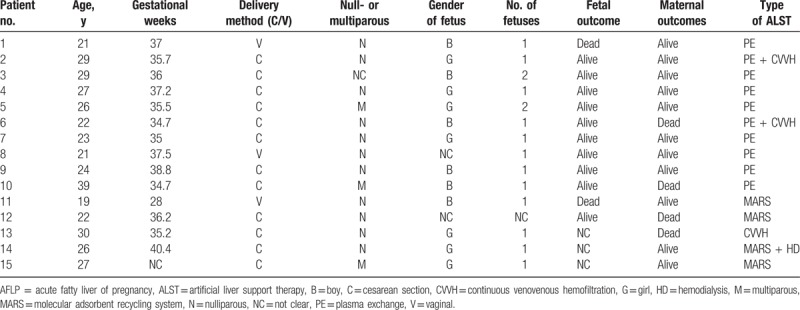
Clinical characteristics of AFLP patients in our hospital.

The average time interval between delivery and start of ALST was 2.7 days (range 0–9 days). Depending on the status and progression of the disease, each patient received various number of ALST sessions (average: 3.1 times/patient, range: 1–11 times). Eventually 4 patients died and the rest were discharged recovered (Table 1), so the overall maternal fatality rate was 26.7%, which is much lower than that before the clinical application of ALST.

### 3.2 Literature search and study selection

A total of 29 potentially relevant studies were identified in PubMed, and 9 studies were eventually included in the systematic review after exclusion of nonrelevant studies and those not meeting the previously mentioned inclusion criteria. We did not find any relevant studies in ClinicalTrials.gov, Embase, and Cochrane. A summary of included studies is shown in Table 2 and a total of 89 patients were included for analysis.

**Table 2:**
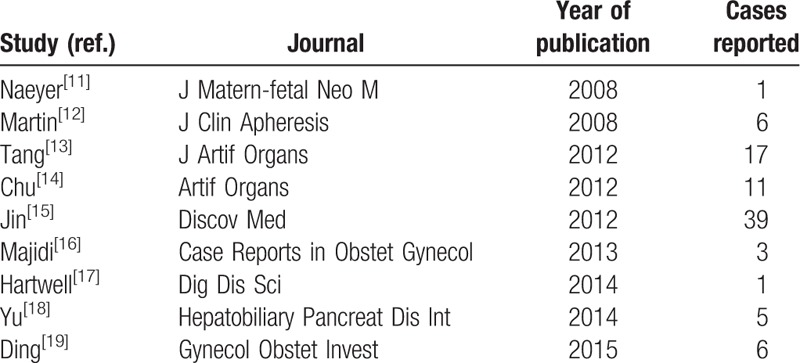
Summary of reported studies included in the review.

### 3.3 Characteristics of the AFLP patients from the chosen studies

The clinical characteristics of the AFLP patients from the chosen studies were summarized in Table 3, and their uses of ALST were summarized in Table 4. Briefly, the mean age of these patients was 26.7 years old (range 20–40), and their mean gestational weeks were 33.9 (range 26–41). Most of the patients were primigravidas and had cesarean sections. Varieties of complications associated with AFLP were reported in these patients including hepatic encephalopathy, disseminated intravascular coagulation (DIC), postpartum hemorrhage, etc. The time interval between delivery and ALST ranged from within 6 hours to 9 days after delivery in different studies. With the reported number we can calculate from these studies, 55% of patients received PE, 20% of patients received PE + CVVH, and 14% of patients received continuous hemodiafiltration. For the rest of the patients, 1 received MARS, 2 patients received PE + continuous renal replacement therapy (CRRT), and 3 patients received PE and PE + CRRT during hospitalization. There were some patients from Ding's study of unknown number receiving PE + plasma perfusion.^[6]^ The number of ALST sessions performed in different studies ranged from 2 to 22 sessions for a single patient. At the end, 5 patients died and the rest 84 patients survived. For the fate of the fetuses, 1 study did not report the status of the fetuses, 1 study reported a dead fetus, and the rest studies all reported live fetuses, but the total number of live fetuses were unknown.

**Table 3:**
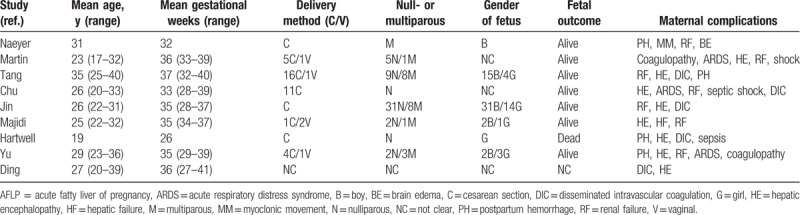
Clinical characteristics of the AFLP patients in reported studies.

**Table 4:**
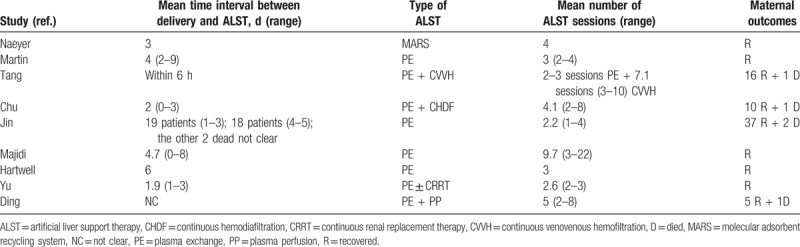
ALST and maternal outcomes in reported studies.

### 3.4 Factors influencing disease outcome

To address the aforementioned questions, associations between: pretreatment total bilirubin (TB) and creatinine (Cr) levels; maternal complications; time interval between delivery and ALST; type and number of ALST sessions used; and maternal outcomes were analyzed with all available studies. Via univariate analysis, we found that only complications, specifically multiple organ dysfunctions (MODS) and postpartum hemorrhage that is defined as the loss of more than 500 mL of blood within the first 24 hours after childbirth were statistically associated with the outcomes of the patients (*P* = .002 and *P* = .032, respectively) while other complications such as infection and coagulopathy were not (Table 5). We also found that neither the time interval between delivery and ALST nor the type and number of ALST sessions was statistically linked to patient outcome (*χ*^2^ = 0.358, *P* = .836; *χ*^2^ = 1.224, *P* = .542; *χ*^2^ = 2.195, *P* = .533, supplemental data, Tables 1–3). By logistic regression analysis, MODS was further found the only factor affecting the final outcome of the patients (recovered or deceased) (odds ratio = 49.8, *P* = .002, Table 6). Surprisingly, we found preoperative serum TB and Cr levels have very little impact on disease outcome (supplemental data, Table 4).

**Table 5:**
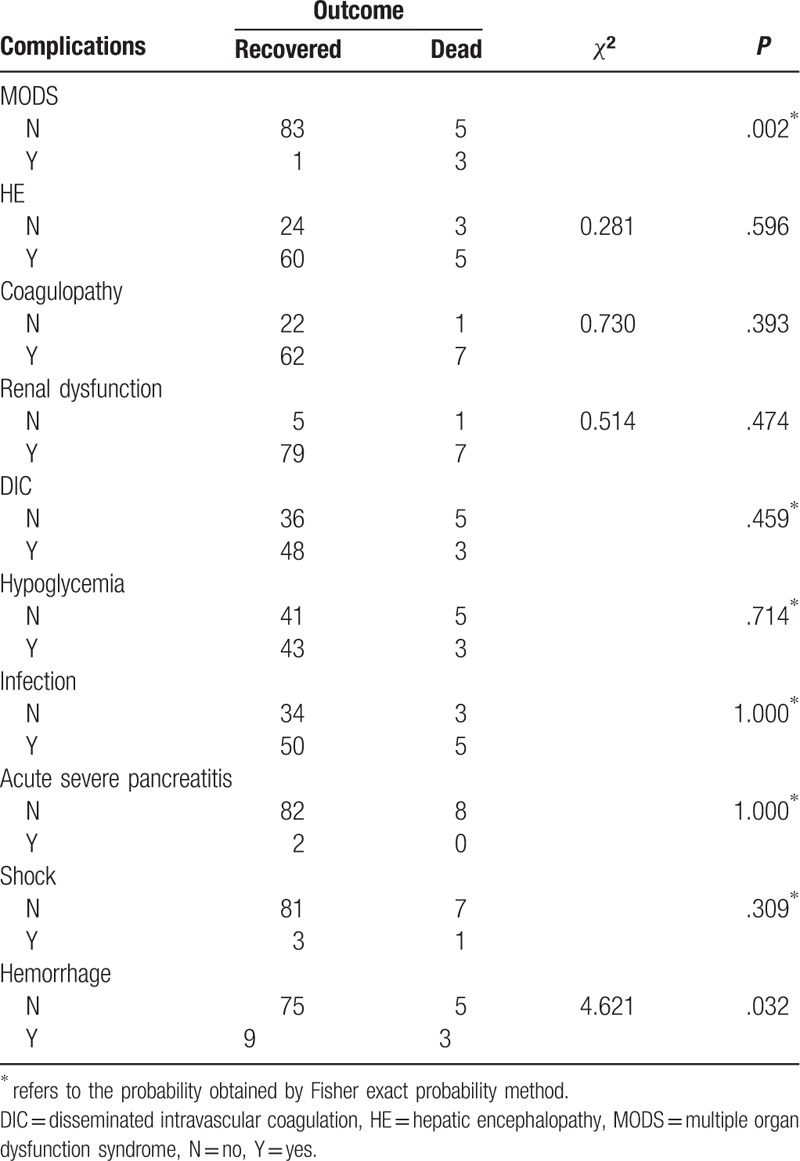
Relationship between complications and disease outcome (n = 92).

**Table 6:**
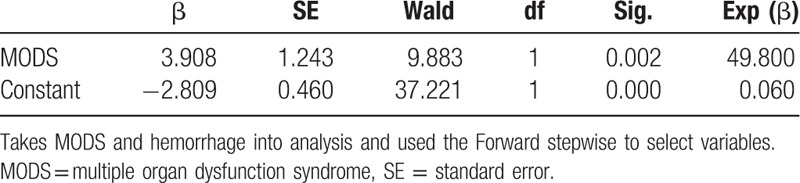
Logistic regression analysis identifying the independent factor affecting disease outcome (n = 92).

## 4 Discussion

AFLP, a serious liver disease occurring mainly in the third trimester of pregnancy and characterized by jaundice and coagulopathy, usually presents with the symptoms of hepatic and possibly renal function impairment. In patients in a critical condition, other severe complications can also be seen such as DIC, hepatic encephalopathy, hypoglycemia, MODS, etc.^[18]^ Pathogenesis of AFLP is associated with acute hepatic failure subsequent to massive necrosis of hepatocytes resulting in accumulation of various toxins, reduced synthesis of essential factors, and endotoxin-induced cytokine storm. All these factors contribute to the development of multiple-organ dysfunction.^[19]^ Besides, quick onset, rapid progression of the disease and being difficult to treat also jeopardize the survival of both mother and fetus.

Thus, how to reduce maternal mortality has long been an issue in clinical management of AFLP. As an in vitro mechanical and chemical device that can temporarily replace part of liver function, ALST has been widely used in the management of acute or chronic liver failure caused by various etiologies,^[10]^ which, to some extent, alleviates liver injury and provides a homeostatic environment for hepatocyte regeneration. Currently available ALST includes hemoperfusion, PE, continuous renal replacement therapy, MARS, etc. As current ALST can only temporarily fulfill the detoxification function of liver and is an auxiliary treatment modality, clinical improvement may only be expected when ALST is used in combination with liver protection and etiology treatments.

In recent years, ALST-including treatment strategy has saved many patients from AFLP induced liver failure. Among the 15 AFLP patients who underwent ALST in our hospital, 11 recovered and 4 died, and the mortality rate was 26.7%. Among the AFLP patients who underwent ALST in the chosen studies, 84 recovered and 5 died, and the mortality rate was 5.6%. Both mortality rates are significantly lower than that before the advent of ALST, which was about 85% in the 1980s,^[14]^ suggesting that ALST does improve the survival of AFLP patients. So far, published data on the outcome of the patients with AFLP are largely based on retrospective studies from small case series. There are no large clinical trials comparing different treatment strategies with patient outcomes, and it is also very unlikely to be feasible to conduct these large trials due to disease scarcity.^[11]^ But certain questions still need to be addressed. These questions include: When should ALST start after delivery to improve the survival of both mothers and infants? How many sessions of ALST should be carried out? Is the type of ALST chosen important? and What factor(s) contribute to the outcome of patients undergoing ALST?

To address above questions, we analyzed the AFLP cases undergoing ALST from our own hospital and selected studies in the literature, and found that only complications, specifically, MODS and postpartum hemorrhage were statistically associated with patient outcome. Further analysis revealed that MODS was the only factor independently affecting patient outcome. Other factors including preoperative serum TB and Cr levels, time interval between delivery and ALST, ALST type and the number of ALST sessions were found not significantly related to patient outcome. We think that 2 possible reasons can explain these results: majority of AFLP patients survived with ALST-including treatment strategy, and the fact that MODS occurred in those demised patients also makes the type, timing, and number of ALST sessions contribute little to the outcome. Previous studies have indicated that occurrence of complications in AFLP would affect disease outcome,^[20]^ but it is unknown whether ALST can improve the outcome of patients with different complications, especially those with MODS. The pathogenesis of MODS in AFLP has not yet been fully elucidated. It is thought that MODS may be related to fatty acid metabolism disorder due to hormone level change in late pregnancy causing accumulation of large amounts of free fatty acids in liver, kidney, brain, and other organs and subsequent multiple-organ dysfunction.^[21]^ Results from other studies suggested that MODS be caused by excessive production of cytokines that were stimulated by endotoxemia in liver failure.^[6]^ MODS is fatal and the mortality rate is 80% to 90%.^[9]^ Our results indicate that ALST cannot improve the prognosis of MODS. Therefore, early intervention and prevention of MODS is essential in the management of AFLP.

It is worth of mentioning that some adverse reactions were observed/reported in certain patients having ALST. Some of our patients developed rash during the course of ALST, but the rash subsided after dexamethasone treatment. Other adverse reactions including acute pulmonary edema, bleeding, hypotension, fever, and hypocalcemia were reported in patients undergoing PE and CVVH.^[13]^ But these adverse reactions occur infrequently and are mild, and are insufficient to warrant discontinuation of ALST in AFLP management.^[11,13]^

Our study also has some limitations. These include small number of published studies found in the public databases and incomplete retrieval of some information from certain selected studies. These limitations may to some extent affect the interpretation of our results, but considering very few cases of AFLP in clinic and our study so far being the only one investigating aforementioned questions, the results of our study provide important information on how to further improve the disease outcome with the implementation of ALST in clinical practice.

In summary, in current study we investigated several key questions regarding the use of postpartum ALST in AFLP management via the method of systematic review. We found that ALST improved the outcome of AFLP patients although more properly designed experiments are needed to confirm this point, and MODS was the main independent factor affecting patient outcome even in ALST era. For that end, our study provides more knowledge on the clinical management of AFLP.

## Author contributions

**Conceptualization:** Wei Zou, Yi Gong, Junhui Wan.

**Data curation:** Zhenping Wu, Wei Zou, Peng Huang.

**Validation:** Peng Huang, Wei Zou.

**Writing – review & editing:** Zhenping Wu, Wei Zou, Peng Huang, Yi Gong, Junhui Wan.

Wei Zou orcid: 0000-0003-1274-7534

## Supplementary Material

Supplemental Digital Content
