# Supplementary material for: Treating acute fatty liver of pregnancy with artificial liver support therapy: Systematic review
Source: Medicine (Baltimore). 2018 Sep 21;97(38):e12473. doi: 10.1097/MD.0000000000012473 (PMC6160087; doi:10.1097/MD.0000000000012473)
Supplement: Supplemental Digital Content [file medi-97-e12473-s001.doc]

Supplemental data Table 1. Relationship of the time interval between delivery and ALST and disease outcome (n=42)

| Time interval  (days) |  | Outcome | | | χ2 | *P* |
| --- | --- | --- | --- | --- | --- | --- |
|  |  | Recovered |  | Dead |  |  |
| 1 |  | 17 |  | 2 | 0.358 | 0.836 |
| 1～3 |  | 16 |  | 2 |  |  |
| 3～ |  | 4 |  | 1 |  |  |

Supplemental data Table 2. Relationship between ALST type and disease outcome (n=91)

| ALST Type |  | Outcome | | | χ^2^ | *P* |
| --- | --- | --- | --- | --- | --- | --- |
|  |  | Recovered |  | Dead |  |  |
| MARS |  | 4 |  | 1 | 1.224 | 0.542 |
| PE |  | 48 |  | 4 |  |  |
| PE+CRRT |  | 32 |  | 2 |  |  |

Supplemental data Table 3. Relationship between the number of ALST sessions and disease outcome (n=91)

| Number of ALST sessions |  | Outcome | | | χ^2^ | *P* |
| --- | --- | --- | --- | --- | --- | --- |
|  |  | Recovered |  | Dead |  |  |
| 1～3 |  | 53 |  | 4 | 2.195 | 0.533 |
| 4～6 |  | 19 |  | 1 |  |  |
| 7～9 |  | 9 |  | 2 |  |  |
| 10～ |  | 3 |  | 0 |  |  |

Supplemental data Table 4 Relationship between pre-treatment serum TB and creatinine levels and disease outcome (n=36, x ± s)

| Variable |  | Outcome | | | *t* | *p* |
| --- | --- | --- | --- | --- | --- | --- |
|  |  | Recovered (n=32) |  | Dead (n=4) |  |  |
| The level of TB before ALST (umol/L) |  | 300.34±213.82 |  | 248.30±144.38 | 0.47 | 0.641 |
| The level of creatinine before ALST (umol/L) |  | 204.43±73.55 |  | 292.25±156.32 | -1.108 | 0.345 |
